# Protocol for Investigating the Technical Efficiency of District Hospitals in the Public Health Sector of KwaZulu-Natal, South Africa

**DOI:** 10.2196/12037

**Published:** 2019-03-14

**Authors:** Tesleem K Babalola, Indres Moodley

**Affiliations:** 1 Department of Public Health University of KwaZulu-Natal Durban South Africa

**Keywords:** technical efficiency, district hospitals, data envelopment analysis

## Abstract

**Background:**

The central objective of policy makers and health managers is efficiency in the delivery of health care. With frequent reports of global economic crises, there is a need to continuously measure the performance of various sectors of the health care system. This can inform the decision-making process toward allocating scarce resources with the aim of maximizing output.

**Objective:**

The aim of this study is to determine the technical efficiency (TE) of public sector district hospitals in the province of KwaZulu-Natal, South Africa to provide information that will assist in policy formulation that may further assist in more efficient resource allocation decisions.

**Methods:**

This is a health system research based on a quantitative research approach. All 38 public district hospitals in the 11 municipalities of the province will be included in this study. The data for the study will include inputs from hospitals’ operations that contribute toward subsequent outputs. The input data will include information such as the number of health professionals (doctors, nurses, and other personnel) and number of hospital beds, whereas the output data will include information such as outpatient visits and number of admissions or discharge. Other data categories to be included will be determined by data availability and will be uniform for all facilities. Data for each facility for a 3-year period from 2014 to 2017 will be obtained from databases of the district health information, basic accounting, and personnel salary systems. On the basis of the data obtained, a model will be developed that can be used to assess how TE of public districts hospitals may be improved. TE will be determined using Data Envelopment Analysis, and factors influencing efficiency will be computed using StataCorp statistical package.

**Results:**

As of February 2019, the study is at the data collection, data input, and analysis stages. The results are expected to be available from the second quarter of 2019.

**Conclusions:**

Findings from this study can add to tools available to policy makers, health planners, and managers in making decisions about resource allocation in health care systems. Moreover, these findings will be disseminated electronically and in print.

**International Registered Report Identifier (IRRID):**

DERR1-10.2196/12037

## Introduction

### Background

*The health of a nation is the wealth of the nation* is a popular saying. Thus, the health sector of any country is critical to social and economic development that links productivity to the quality of health care. According to the Millennium Development Goal (MDG) report of 2015, most sub-Saharan African countries failed to achieve the health-related targets set by the United Nations [1]. The health-related goals of MDG include reducing child mortality, improving maternal health, and combating HIV/AIDS, malaria, and other diseases, which represent the major health challenges in South Africa (SA) and other sub-Saharan African countries [1]. There is a need to continuously monitor the efficiency of health care facilities, especially the district hospitals, which offer generalist services and support the basic primary health care service. One of the goals of the newly introduced 2030 agenda of the United Nations known as *Sustainable Development Goals* is ensuring healthy living and promoting well-being for all ages [2]. Consequently, periodic monitoring of health care service delivery at health facilities is necessary.

Sub-Saharan Africa accounts for 11% of the world’s population, yet it bears 24% of the global disease burden and allocates less than 1% of global health expenditure [3]. In this region, there is also a severe shortage of trained medical personnel, with just 3% of the world’s health workers deployed in sub-Saharan Africa [3]. Reports have shown that sub-Saharan African countries are faced with a heavy burden of both communicable and noncommunicable diseases [1,3]. Unfortunately, most of these countries (especially areas with the greatest health care needs) lack adequate information for health planners to better understand and address problems related to equitable distribution of health care services. A report shows that the region of sub-Saharan Africa spent an average of 6.1% of its total gross domestic product (GDP) on health, far less than the 9.5% of GDP that countries of the Organization for Economic Co-operation and Development (OECD) spend on health [4]. However, SA on the other hand spends 8.8% of its GDP on health, which is higher than the health-related expenditure of most other sub-Saharan African countries and closer to OECD countries [4].

The mission of the SA National Department of Health (DoH) is to improve the health status of the population through prevention and health promotion [5] and also to consistently improve the health care delivery system by focusing on access, equity, efficiency, quality, and sustainability [5]. In 1994, the democratically elected government inherited a fragmented health system that was characterized by inequitable access, distribution, and financing [6]. In an attempt to redress the imbalances of the past, the DoH embarked on reforms as articulated in the white paper on transformation of the health system [6]. To improve the health status, the government, through the DoH, adopted the district health system as the operational vehicle to deliver comprehensive primary health care, of which district hospitals are an integral part. The district hospital plays a pivotal role in supporting the primary health care, and it is also the gatekeeper to more specialist care at provincial and tertiary hospitals [6].

The central objective of policy makers and health managers is to ensure efficiency in the delivery of health care [7]. For this reason, there is a growing interest in the measurement of inputs, activities, and outcomes of health systems. This is because of an increase in the cost of health care, increased demand for public accountability, and improved capabilities for measuring system performance. Where high levels of technical inefficiency exist, there is a significant concomitant waste of available resources. Even though efficiency occupies a central role in health policy, much of the attention of policy makers, donors, and health care researchers has been on health sector reforms and the mobilization of additional resources to redress inequalities in access to health care [8]. Given the level at which resources are being mobilized for health care services, it is important to investigate the efficiency with which these resources are used.

Hospitals are known to absorb the greatest proportion of the total health expenditure in most sub-Saharan African countries, estimated at over 45% to 69% of government health sector expenditure [9,10]. The SA health care system comprises a large public health sector that consumes around half of the 9% of the total expenditure and is collectively higher than 5% of the GDP recommended by the World Health Organization [11]. Despite the high expenditure, the country’s health outcomes are poor in comparison with other similar middle-income countries, reflecting inequity in health care in the country [11]. The public hospital sector accounted for a very high proportion (80.9%) of the total health expenditure in 2016 and 2017. More than half (54.6%) of the total hospital expenditure in 2016 and 2017 was consumed by district hospitals [12]. As hospitals are increasingly consuming more health care resources, there is a need to determine if the increase is accompanied by increase in service provision.

Therefore, it is important to evaluate the efficiency of hospitals, as failure to do so will compromise efforts toward redressing inequities and access to health care. Improving the efficiency of hospitals is central to the overall improvement of health system performance as it will enable the redistribution of potential resources to ensure equity, accessibility, and the delivery of sustainable quality care.

### Aim and Objectives

The overarching aim of this study is to determine the technical efficiency (TE) of public sector district hospitals in the province of KwaZulu-Natal (KZN), SA to provide analysis to inform policy formulation that may enable more efficient resource allocation decisions. The following objectives are identified to address the aim of the study. Firstly, to assess different approaches toward measuring TE of health facilities using a systematic review. Secondly, to determine the TE level of the selected district hospitals. Thirdly, to estimate the adjustment needed to make inefficient facilities more efficient. Fourthly, to identify factors that influence the performance and efficiency of these hospitals. Fifthly to compare the TE of rural district hospitals to those in urban areas. Finally, to develop a model and framework to provide recommendations for improving efficiency of SA district hospitals.

## Methods

### Overview

KZN is the second most populous province in SA with a total population of above 10 million, comprising more than 85% black Africans. It is the largest province located at the southeastern part of the country, comprising 11 districts and 52 municipalities, which are a mix of urban, semi-urban, and rural areas. It is bordered on the east by the Indian Ocean and other parts by 3 other provinces and 3 other Southern African countries: Mozambique, Swaziland, and Lesotho [13].

There are 3 categories of hospitals in the country: the district, regional, and tertiary (provincial tertiary and national central) hospitals. District hospitals account for 64% of public hospitals in the country. It is the first level of referral, and it provides generalist health care where various outpatient and inpatient services are offered. District hospitals have between 50 and 600 beds, a 24-hour emergency service, and an operating theater. Specialists from different clinical services provide a range of diagnostic, therapeutic, and rehabilitative services [14].

### Sample Size and Sampling Strategy

The sample size for this study will be all 38 public district health hospitals (DH) in KZN in the 11 municipalities of the province listed in Table 1. Any DH without available/retrievable data from the national database will be excluded from the study.

### Study Design

This is a health system research based on a quantitative research approach to determine the TE of district hospitals in the KZN province using Data Envelopment Analysis (DEA). DEA was first introduced by Charness et al in 1978 for measuring the relative efficiency of organizations such as hospitals and schools [15]. DEA (a nonparametric method) defines efficiency as the ratio of the weighted sum of outputs of an organization to its weighted sum of inputs. It is particularly useful in public sector organizations (eg, health facilities) that lack the profit maximization motive and employ multiple-input and multiple-output production processes [15].

DEA uses linear programming techniques to compute the efficiency scores. Facilities that are technically efficient have a score of 1 or 100%, whereas inefficient hospitals have efficiency scores of less than one (ie, less than 100%) [15]. Efficiency of an organization is measured relative to an observed best practice within a set. This indicates that the benchmark against which to compare the efficiency of a district hospital is determined by the group of hospitals in the study and not a value fixed by hospitals outside of the group [16].

**Figure 1:**
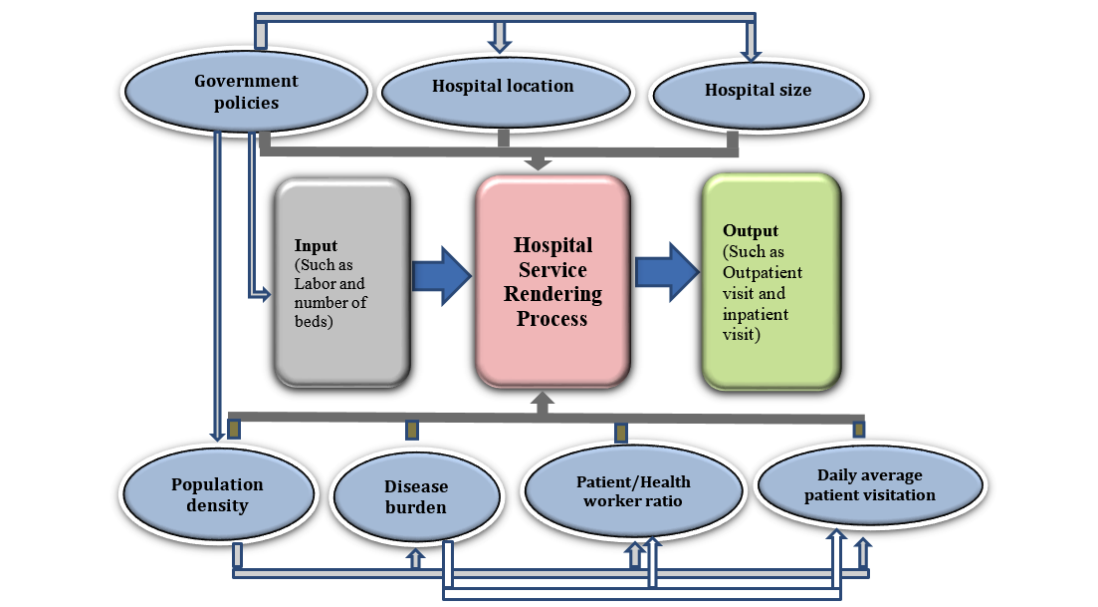
A theoretical framework for hospital technical efficiency (designed by the researchers).

Some of the positive characteristics of DEA are that it can handle multiple-input and multiple-output models, it does not require an assumption of a functional form relating inputs to outputs, the facilities are directly compared against a peer or combination of peers, and finally, input and output variables can have different measuring units [16]. The theoretical framework for this study is as shown in Figure 1.

#### Data Types to Be Explored

The required data for this study will relate to direct services provided to patients at district hospitals and the inputs employed by the health facility to generate services and outputs, which reflect the general scope of the facility’s health care activities.

Improved health status is the ultimate output of hospitals or the health system at large. However, because of difficulties in accurately measuring improvements in health status, hospital output is measured by an array of intermediate health services that are surrogate markers of changes in health status. The selection of inputs and outputs for a DEA study requires careful thought as the distribution of efficiency is likely to be affected by the definition of outputs and the number of inputs and outputs included [17].

There are 2 major views toward defining and measuring the output of health care organizations [18]: first, *the process approach* asserts that the output of a health care organization comprises services provided by the different units such as the radiology, laboratory procedures, patient days, etc. Second, *the outcomes approach* regards the above processes only as intermediate steps leading to the desired change in a patient’s health status, that is, output should be measured in terms of the end result or outcome, that is, improved health [18]. Though the generally agreed opinion is to measure health care output through improvement in service quantity and quality of life, it is easier to measure and define services rendered than changes in health status [16]. Health is multidimensional and is affected significantly by a host of other socioeconomic factors. Thus, output is measured as a range of intermediate outputs (health services) that purportedly improve health status. Therefore, the choice of data selection will be made on the basis of data availability and the input and output variables used in previous health care efficiency studies in Africa.

Inputs in hospital production are classified as labor, capital, and supplies. In most studies, the number of hospital beds is a proxy for capital. Thus, in this study, the input variables will focus on the number of health care professionals (doctors, nurses, and other personnel) and the actual number of hospital beds. More focus will be based on health professionals who are directly linked with health care provision. Information on the hospital expenditure within the periods under study will also be retrieved. On the other hand, hospital outputs for the DEA model will be identified from the district health information system (DHIS) database. This will include information such as admissions, outpatient visits, inpatient days, and number of admissions/discharges. Data type to be included will be determined by data availability, and it will be uniform for all facilities.

**Table 1:** List of district hospitals in KwaZulu-Natal, by municipality.

Municipality	District hospitals
Amajuba	Niemeyer Memorial Hospital
eThekwini	Osindisweni Hospital
	St Mary’s Hospital
	Wentworth Hospital
Harry Gwala	Christ the King Hospital
	EG Usher Mem Hospital
	Rietvlei Hospital
	St Apollinaris Hospital
iLembe	Montebello Hospital
	Umphumulo Hospital
	Untunjambili Hospital
Ugu	GJ Crooke’s Hospital
	Murchison Hospital
	St Andrew’s Hospital
uMgungundlovu	Appelsbosch Hospital
	Northdale Hospital
Umkhanyakude	Bethesda Hospital
	Hlabisa Hospital
	Manguzi Hospital
	Mosvold Hospital
	Mseleni Hospital
Umzinyathi	C Johnson Mem Hospital
	Church of Scotland Hospital
	Dundee Hospital
	Greytown Hospital
Uthukela	Emmaus Hospital
	Estcourt Hospital
Uthungulu	C Booth Hospital
	Ekhombe Hospital
	Eshowe Hospital
	KwaMagwaza Hospital
	Mbongolwane Hospital
	Nkandla Hospital
Zululand	Benedictine Hospital
	Ceza Hosp
	Itshelejuba Hosp
	Nkonjeni Hosp
	Vryheid Hosp

#### Data Collection Technique

The data collection technique for this study will be a consecutive sampling of all the district hospitals in the province as they occur in the national health database. On the basis of data availability, input and output data for a 3-year period between 2014 and 2017 will be retrieved. Nonfinancial input and output data will be obtained from the DHIS database and financial data (input data) will be obtained from the basic accounting system and policy on the personnel salary system, with permission from the KZN provincial DoH.

However, to obtain information on the likely factors that affect the performance of hospitals, some sets of information, which are not directly linked to delivery of health care services such as population density, disease burden, facility location, patient/health workers ratio, and daily average patient visitation, as contained in the database, will be retrieved.

#### Measures to Ensure a Scientifically Rigorous/Trustworthy Study

This will be done by making sure that selection for both input and output variables will be guided by previous health care efficiency studies and data availability within the database. The variables that will be chosen will be adequate to cover the general activities at the district health facilities. Data validity and reliability check will be done by randomly visiting some of the district hospitals for discrepancy check and data confirmation.

#### Proposed Data Analysis

The data obtained for the stated variables will be entered in Microsoft excel spreadsheets. As this study includes identifying the sources and magnitude of possible inefficiency in the health care system, it demands the use of DEA. TE analysis will be carried out using the DEA software package. The combination of the efficiency measurement system and open-source DEA software programs will be used in computing the DEA efficiency scores.

Each facility represents a decision-making unit (DMU), which is also sometimes referred to as data management unit in DEA. The DEA program requires that the data should be listed by observation, that is, each row for a DMU. The input and output variables will be listed in the column.

The frontier against which the TE of all hospitals is measured is defined by those hospitals in the group with a TE score of 100%. The hospitals producing on the efficient frontier define the best practice and thus could be regarded as role models. For each inefficient hospital, the DEA model has identified efficient hospitals that could be used as comparators [16]. The inefficient hospitals are expected to learn from their efficient peers by observing their production process [16].

Correlation and regression analysis will be computed to determine factors influencing efficiency using StataCorp statistical package.

#### Data Management and Storage Plan

The data obtained and entered in Microsoft excel spreadsheets will be stored in a back-up hard drive and in a protected cloud storage for safe and easy retrieval in the future. Data will be kept confidential during the study and in secure storage for 2 years after study completion. All personal data and details will be deleted, and no identifying information will be published.

#### Data Envelopment Analysis Model for Estimating Technical Efficiency

**Figure 2:**
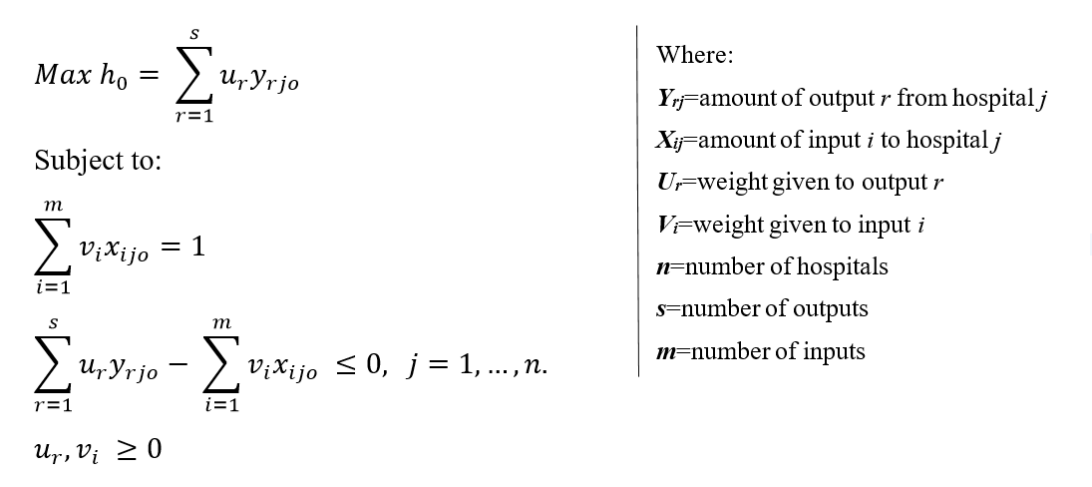
Data envelopment analysis model proposed by Charness et al.

Charness et al proposed a constant return to scale for the DEA linear programming model, which stated that an increase in input should result in a proportionate increase in output [15]. The model is illustrated in Figure 2.

### Ethics Approval and Consent to Participate

Ethical approval was obtained from the Ethics Committees of the University of KwaZulu-Natal (HSS/0805/017D) and KwaZulu-Natal DoH (HRKM301/17). As this study did not directly involve human participants, instead of participants consent, permission to access the provincial health database was sought from the KZN health district manager.

## Results

As of February 2019, the study is at the data collection, data input, and analysis stages. The study timeline in Table 2 illustrates that the results are expected to be available from the second quarter of 2019.

**Table 2:** Proposed study timeline where Q indicates quarter.

Year	2017			2018				2019			
Task	Q2	Q3	Q4	Q1	Q2	Q3	Q4	Q1	Q2	Q3	Q4
Preparation of detailed research proposal and ethical approval	✓^a^	✓	✓								
Systematic and literature reviews			✓	✓	✓	✓	✓	✓			
Field work/data collection					✓	✓	✓	✓			
Data input/data analysis							✓	✓	✓		
Dissemination of study findings									✓	✓	✓

^a^✓: period of activity.

## Discussion

It is vital to assess the TE of district and other hospitals to be able to utilize the available resources optimally and expedite the move toward achieving health and development goals. Findings from this study can add to the tools available to policy makers, health planners, and managers in making decisions about resource allocation in the health care system.

### Dissemination of Study Findings

The aim of this study is to add to the tools available to policy makers, health planners, and managers in making decisions about the allocation of limited health care resources with the aim of maximizing the output from the health care system.

Findings of this research will be made available through publications in internationally peer reviewed journals and presentations at both local and international conferences. This will create information access for policy makers, health planners, and managers in making decisions about the allocation of health care resources with the aim of maximizing the output from the health care system. Moreover, the report of findings will be made available to KZN DoH.

### Strengths and Limitations of the Study

Multiple hospital inputs and outputs will be used in computing efficiency as against the usual efficiency measurement through direct single input and output relationship. Due to time and cost constraints, the study could not cover all provinces in the country and as such, findings from this study may not be considered fully representative of the overall national situation. Limitations related to DEA approach can also have an effect on the study. These include the following: (1) Inability to compare the TE with district hospitals from other provinces in SA as DEA measures the efficiency relative to the best practices within hospitals in a sample group, (2) the result is sensitive to measurement error, that is, an outlier because of an inflated hospital input or output can significantly reduce the efficiency of other hospitals.

## Abbreviations

DEA: Data Envelopment Analysis
DH: District hospitals
DHIS: District health information system
DMU: Decision-making unit
DoH: Department of Health
GDP: Gross domestic product
KZN: KwaZulu-Natal
MDG: Millennium Development Goal
OECD: Organization for Economic Co-operation and Development
SA: South Africa
TE: Technical efficiency
